# 

**Published:** 1887-02

**Authors:** 


					﻿OLD SERIES
Vol. xxv
<7 1855 4
NE> 'SERIES
Vol. HI.—No. 12.

v by* W.F.WESTMOREEANDJW^
JA H.V.M.MILLER.M.D.LL.D.JRL
t/V**	JAMES A.GRAY, M.D.'^A
Published By james p.harrison&cg|
fl;______________Atlanta >ga. Jm
SUBSCRIPTION: $2.60 A YEAR.
				

